# The Prevalence of Clinically Undiagnosed Depression in Patients With Autoimmune Bullous Diseases Seen at Inkosi Albert Luthuli Central Hospital, Durban, South Africa

**DOI:** 10.7759/cureus.54610

**Published:** 2024-02-21

**Authors:** Nkosiyenzile Cele, Josiah T Masuka, Khumo Duze, Anisa Mosam

**Affiliations:** 1 Department of Dermatology, School of Clinical Medicine, College of Health Sciences, University of KwaZulu-Natal, Nelson R Mandela School of Medicine, Durban, ZAF; 2 Department of Medicine, Faculty of Health Sciences, University of Zimbabwe, Harare, ZWE

**Keywords:** psychiatric comorbidity, bullous dermatoses, major depressive disorder (mdd), autoimmune bullous disease, psychodermatology

## Abstract

Background

Chronic autoimmune bullous diseases have been associated with major depression in previous studies. This has been attributed to inflammatory cytokines, chronic pain, and the chronicity and debilitating nature of the disease. As no similar studies have been conducted in our setting, we aimed to determine the prevalence and severity of clinically undiagnosed depression in patients with autoimmune bullous diseases.

Methodology

We performed a cross-sectional study among outpatients managed in a bullous disease clinic at Inkosi Albert Luthuli Central Hospital, a quaternary provincial hospital in Durban, South Africa.

Results

A total of 44 participants were recruited and included in this study. The majority of the participants were females (29, 65.9%). The most common autoimmune bullous diseases were pemphigus vulgaris (19, 43.2%), bullous pemphigoid (18, 40.9%), and pemphigus foliaceus (5, 11.4%). The overall prevalence of at least mild and at least moderate depression in patients with autoimmune bullous diseases in our clinic was 52.3% and 20.5%, respectively. Pemphigus vulgaris showed the highest median Patient Health Questionnaire-9 score compared to other bullous dermatoses. Statistically significant differences were observed between females and males for the duration with the bullous disease (p = 0.014) and between intraepidermal and subepidermal disease for both the mean age (p = 0.038) and age at onset (p = 0.015).

Conclusions

Clinically undiagnosed depression is common in patients with autoimmune bullous disease. Its frequency and severity may differ depending on the underlying autoimmune bullous disease and possibly other factors. Dermatologists should always be alert to this fact and prompt psychiatric consultation as required to comprehensively manage these patients.

## Introduction

Bullous diseases are blistering eruptions of the skin and/or mucous membranes which are classified as either autoimmune or non-autoimmune dermatoses. They account for 0.5% and 19.5% of all dermatology outpatients in South Africa [1] and Sudan [2], respectively. In the Western Cape province of South Africa, bullous diseases account for 5.3% of all admitted dermatology patients [3], indicating an increased burden of these dermatoses to the dermatology workforce. Furthermore, bullous diseases are a major cause of significant morbidity and mortality, accounting for 57.8% of deaths from all dermatological admissions [4].

Bullous dermatoses have also been associated with psychiatric diseases such as anxiety and/or major depressive disorders (MDDs) among other conditions. They significantly impact an afflicted patient’s psychological health and quality of life, possibly due to the disfiguring cutaneous lesions, functional challenges, and disease chronicity [5]. However, psychiatric complications such as MDD, defined as a pervasive lowering of mood, may result from the chronic use of immunosuppressive corticosteroid therapy [6], thus confounding the real association between mood disorders and bullous diseases [7]. Moreover, comorbid chronic diseases, a family history of MDD, alcohol abuse, and socioeconomic challenges are additional confounders [8].

To date, conflicting evidence indicates that bullous disorders such as bullous pemphigoid may predispose to the onset of psychiatric disorders including MDD [9,10], although other studies have indicated contrary results [11]. Moreover, there is no local study characterizing the mental health of patients with autoimmune bullous diseases (AIBDs). Given the lack of local data on this subject [12], we conducted a cross-sectional study to determine the prevalence and severity of clinically undiagnosed depression in patients with AIBDs. We also aimed to compare the levels of depression between different AIBDs in addition to exploring the relationship between the severity of depression and the duration of the AIBD.

## Materials and methods

Study design

We conducted a cross-sectional study among AIBD patients attending a longitudinal bullous dermatosis clinic at Inkosi Albert Luthuli Central Hospital (IALCH). The study was performed over a three-month period from October 2022 to December 2022.

Study setting

IALCH is a quaternary referral and teaching hospital situated in the coastal city of Durban in the KwaZulu-Natal province of South Africa. This hospital caters to referrals of difficult cases from tertiary and other hospitals in the province and provides specialist and subspecialist services under four main domains/disciplines, namely, surgery, medicine, mother and child care, and perioperative services [13].

Inclusion and exclusion criteria

We included all consenting, biopsy-proven AIBD patients in the study using a convenience sampling method for patients attending the IALCH dermatology outpatient clinic. Patients with known major depressive comorbidity were excluded from participation in this study.

Data collection

Two questionnaires were administered to the participants. An interviewer administered the study questionnaire followed by a validated Patient Health Questionnaire-9 (PHQ-9) depression scoring questionnaire to collect each participant’s demographic and clinical data and to evaluate their depression scores, respectively. The validated PHQ-9 scores of 5, 10, 15, and 20 were chosen to represent the lower limits for mild, moderate, moderately severe, and severe depression, as shown by Kroenke et al. [14].

Statistical analysis

Data on the participants’ age, gender, race, education level, employment, medical history, and bullous disease diagnosis and duration were collected using the study questionnaires and then transferred to a Microsoft Excel sheet (Microsoft Corporation, Redmond, WA, USA). The data were then analyzed using SPSS version 22.0 (IBM Corporation, Armonk, NY, USA). Descriptive statistics such as frequencies and percentages were used to summarize categorical variables, while continuous variables were described using either means and standard deviations or medians and interquartile range (IQR) as appropriate. Comparisons of count data were done using the chi-square or Fisher’s exact test as appropriate, whereas comparisons of continuous data were done using the independent Student’s t-test. All analyses were done at the 0.05 significance level.

Ethical considerations

The study was approved by the IALCH ethics committee and the University of KwaZulu-Natal Biomedical Research Ethics Committee (BREC) (approval number: BREC/00004388/2022). Informed consent was obtained from all participants, and the study data was kept confidential.

## Results

Demographic and social characteristics

**Figure 1:**
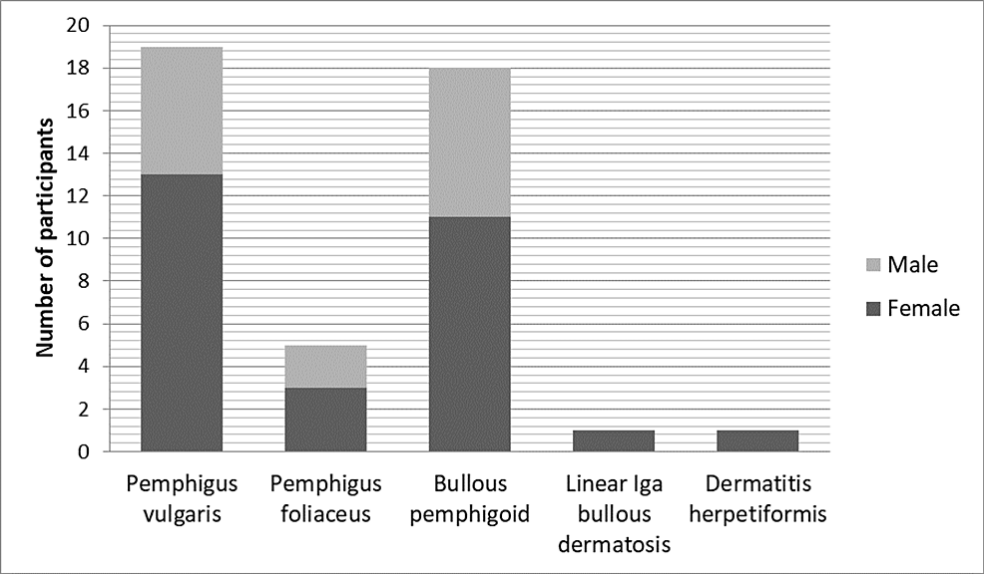
Distribution of autoimmune bullous diseases.

A total of 44 participants were recruited and included in this study. Of these participants, 27 (61.4%) were classified as Indian while the remaining were Black (17, 38.6%) as shown in Table 1. Most participants were females (29, 65.9%). The median age of the participants was 56 (IQR = 40.25-64) years. As highlighted in Figure 1, the most encountered AIBDs were pemphigus vulgaris (19, 43.2%), bullous pemphigoid (18, 40.9%), and pemphigus foliaceus (5, 11.4%) with median ages of 54 (IQR = 36-63), 63.5 (IQR = 56-73.5), and 50 (IQR = 24.5-53.5) years, respectively. A minority of the study participants had achieved tertiary-level education (9, 20.5%), and only 6 (13.4%) reported gainful employment.

**Table 1:** Demographic and social characteristics. PHQ-9: Patient Health Questionnaire-9

Characteristic		Total	Female (N = 29)	Male (N = 15)	P-value
Mean age (years) ± SD		53.43 ± 17.898	56.00 ± 16.403	48.47 ± 20.142	0.189
Age at onset (years) ± SD		49.57 ± 17.054	51.21 ± 15.812	46.40 ± 19.416	0.382
Duration (years) ± SD		4.05 ± 3.785	5.03 ± 4.153	2.13 ± 1.885	0.014
Mean PHQ-9 score		5.70 ± 4.196	6.28 ± 4.242	4.60 ± 4.014	0.213
Ethnicity	African	17	11	6	0.894
	Indian	27	18	9	
Employed	No	38	25	13	0.966
	Yes	6	4	2	
Education level	Primary	13	11	2	0.079
	Secondary	22	11	11	
	Tertiary	9	7	2	
Marital status	Divorced	7	5	2	0.928
	Married	22	14	8	
	Single	15	10	5	

Clinical characteristics

Thirty participants had no reported comorbid disease. Of the remaining 14 participants, hypertension (6, 13.6%) was the most reported comorbid disease. The other comorbid diseases were asthma, pulmonary hypertension, hidradenitis suppurativa, hypothyroidism, iron deficiency anemia, and rheumatoid arthritis. None of the participants reported prior psychiatric disorders. One participant self-reported being HIV positive, while another participant self-reported drug abuse.

**Figure 2:**
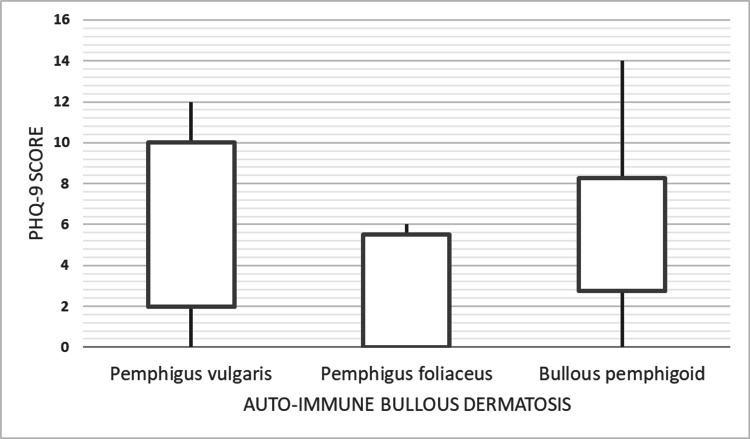
PHQ-9 score distribution for each autoimmune bullous disease. PHQ-9: Patient Health Questionnaire-9

About half of the patients were depressed, as measured by the PHQ-9 questionnaire, with 23 (52.3%) scoring greater or equal to 5 indicating mild depression. Overall, 9 (20.5%) scored greater or equal to 10, indicating moderate depression. Pemphigus vulgaris showed the highest median PHQ-9 score of 6, followed by bullous pemphigoid and pemphigus foliaceus with median scores of 4.5 and 2, respectively. The greatest proportion of participants with PHQ-9 scores greater or equal to 5 was observed for pemphigus vulgaris in 11 out of 19 (57.9%), bullous pemphigoid in 9 out of 18 (50%), and pemphigus foliaceous in 2 out of 5 (40%). The respective IQRs and spread of the PHQ-9 scores for each AIBD are shown in Figure 2. Linear IgA bullous dermatosis (PHQ-9 score = 2) and dermatitis herpetiformis (PHQ-9 score = 13) were excluded from Figure 2 as they had one patient each. Statistically significant differences were observed between females and males for the duration with the bullous disease (p = 0.014) and between intraepidermal and subepidermal disease for the current age (p = 0.038) and age at onset (p = 0.015). The other characteristics did not differ significantly, as shown in Table 1 and Table 2. A total of 15 (34.1%) participants had access to a tub.

**Table 2:** Comparison between intraepidermal and subepidermal autoimmune dermatoses. PHQ-9: Patient Health Questionnaire-9

Characteristic		Total	Intraepidermal blister (N = 25)	Subepidermal blister (N = 19)	P-value
Mean age (years) ± SD		53.43 ± 17.898	48.60 ± 16.373	59.79 ± 18.241	0.038
Age at onset (years) ± SD		49.57 ± 17.054	44.20 ± 15.019	56.63 ± 17.30	0.015
Duration (years) ± SD		4.05 ± 3.785	4.48 ± 4.492	3.47 ± 2.590	0.389
Mean PHQ-9 score		5.70 ± 4.196	5.40 ± 4.203	6.11 ± 4.267	0.587
Ethnicity	African	17	7	10	0.096
	Indian	27	18	9	
Employed	No	38	20	18	0.213
	Yes	6	5	1	
Education level	Primary	13	4	9	0.053
	Secondary	22	16	6	
	Tertiary	9	5	4	
Marital status	Divorced	7	5	2	0.580
	Married	22	11	11	
	Single	15	9	6	

## Discussion

In this study, we set out to determine the prevalence of clinically undiagnosed depression in patients with AIBDs and to describe the characteristics of these patients. The overall prevalence of mild and at least moderate depression in patients with AIBDs in our clinic was 52.3% and 20.5%, respectively. The proportion of patients with at least mild depression and the median depression scores were highest in patients with pemphigus vulgaris, bullous pemphigoid, and pemphigus foliaceus in that order. In addition, females attending the bullous clinic tended to have a statistically significant longer duration with the disease compared to males.

The occurrence of MDD in patients with AIBD, and particularly autoimmune dermatoses, is not surprising. In fact, inflammatory markers such as tumor necrosis factor-alpha and interleukins 6 and 8 have been found to be markedly elevated in both AIBD and major depression [15]. Apart from the potential inflammatory etiopathogenesis, AIBDs reduce an individual’s self-esteem and quality of life leading to psychosocial effects inclusive of depression [16]. Previous studies have indicated that the prevalence of depressive symptoms ranges from 40% to 80%, while that of major depression ranges from 11.4% to 28% [12]. This is significantly lower than the prevalence observed in our clinic. This could be due to differences in the chosen severity level for reporting the prevalence and the different depression assessment tools utilized in the different studies. However, when we consider moderate depression scores, the observed 20.5% prevalence compares favorably with the above range from the systematic review by Pourali et al. [12].

The prevalence of depression appears to vary with the underlying AIBDs, as indicated in the present study. However, this contrasts with a previous study which did not find any statistically significant differences in the prevalence of depression between pemphigus vulgaris and pemphigus foliaceus, for instance [17]. Furthermore, the risk of developing AIBD-associated depression appears to be higher in females, the elderly, and patients with comorbid diseases [18]. However, this finding may be biased as females tend to have higher healthcare service utilization tendencies [19]. Unfortunately, we cannot comment on the effect of comorbid diseases in this study as they appear to be under-reported. The high levels of unemployment in AIBD patients observed in this study may contribute to the observed depression levels. Previous research has consistently indicated increased levels of distress and depression among the unemployed [20]. Other social factors such as social isolation and stigma observed with bullous diseases may increase the psychosocial stress in these patients and lead to depression [16]. Moreover, the lack of certain social amenities such as bathtubs observed in our study may compound the patients’ bullous morbidity and psychosocial well-being. The lack of such amenities and sometimes the required medicines may present extra stressors to the patient.

The lack of a comparator group in this study limited our insights into the magnitude of the impact of AIBDs on depression severity against other dermatoses. In addition, the small sample size of this study further limited its internal and external validity, particularly the exploration of whether there are significant differences in the PHQ-9 scores between males and females for each autoimmune bullous dermatosis. Furthermore, the obtained sample size limited the generalizability of our study findings in our setting. However, this study managed to highlight and suggest different observations in that different AIBDs may have varying susceptibilities to depression unlike previous findings [17]. The study also demonstrates that some patients with AIBDs have depression and that this can be of different severity possibly influenced by the type of AIBD, as indicated in this study, or other factors, as previously discussed by other studies [18].

## Conclusions

In summary, clinically undiagnosed depression is common in patients with AIBD. Its frequency and severity may differ depending on the underlying AIBD and possibly other factors. The underlying type of AIBD as well as the disease chronicity, frequency of recurrences, and the use of corticosteroids may influence the severity of the observed depression. Thus, dermatologists managing patients with AIBD should always be alert to this fact and promptly refer these patients for appropriate psychiatric management. Despite the rarity of AIBD in our setting, the severity of AIBD and MDD warrants further exploration of our study findings using a larger sample through a multicenter study of facilities managing AIBDs within South Africa and/or the region to fully characterize the problem.
